# Antisocial Personality disorder. A case report

**DOI:** 10.1192/j.eurpsy.2022.1141

**Published:** 2022-09-01

**Authors:** V. Muñoz Martinez, A. León-Parente, M.-D. Laura

**Affiliations:** 1 Hospital General Universitario de Ciudad Real, Adolescents Inpatient Unit., Ciudad Real, Spain; 2 Hospital General Universitario de Ciudad Real, Adolescente Inpatient Unit., Ciudad Real, Spain; 3 Hospital General Universitario de Ciudad Real, Psychiatry, Ciudad Real, Spain

**Keywords:** antisocial, personality, inpatient, disorder

## Abstract

**Introduction:**

Antisocial disorder is characterised by difficulty to adapt to social norms that normally rule different aspects of the person’s conduct in adolescence and adulthood. According to DSM-V, this disorder’s prevalence stands between 0.2% and 3%, and is more frequent in men.

**Objectives:**

Numerous studies have been made about the influence between the environment and genetics for the development of this disorder, finding in several patients a punctual mutation of the monoamine oxidase gen (MAOA); although impulsive behaviour has also been associated to the 5-HT tranporte gene (5-HTT), and the protein coding gene for Tryptophan Hydroxylase TPH1

**Methods:**

The hospital admission for these patients must be made when there’s autoregressive or hetero aggressive behaviour, suicide attempts, psychotic symptoms, or symptoms that generate important repercussions in the person’s normal functions. Nevertheless, is important to identify during the hospitalization the improvement possibilities of these patients in order to make drug or psychotherapy adjustments; in the case that we don’t observe treatment benefits, the patient will be released from the hospitalization

**Results:**

The main treatment is psychotherapy.

**Conclusions:**

There’s not much evidence of drug use in this disorder, however, mood stabilizers, antidepressants, atypical antipshychotics and benzodiazepines are used for rage control, impulsiveness, anxiety and aggressiveness.

**Disclosure:**

No significant relationships.

